# Reddish-Brown Dermal Papules and Plaques in a Patient With Kidney Stones

**DOI:** 10.7759/cureus.85076

**Published:** 2025-05-30

**Authors:** Abhishek P Mullapudi, Lydia A Luu, Mary Noland

**Affiliations:** 1 Department of Dermatology, University of Virginia, Charlottesville, USA; 2 Department of Dermatology, University of Minnesota, Minneapolis, USA

**Keywords:** clinical rheumatology, cutaneous manifestations, cutaneous sarcoidosis, dermatology, extra-pulmonary manifestations of sarcoidosis, hydroxychloroquine, multisystemic sarcoidosis, pulmonary sarcoidosis, rheumatology, systemic inflammatory and autoimmune disease

## Abstract

Sarcoidosis is a systemic inflammatory disease affecting multiple organs including the skin, lungs, and kidneys. This case report describes a man in his late 20s presenting with recurrent kidney stones and a one-year history of progressive, asymptomatic lesions on his scalp, forehead, trunk, and feet, with dyspnea on exertion. Biopsy confirmed cutaneous sarcoidosis, and further evaluation revealed pulmonary and renal involvement. He was treated with hydroxychloroquine and prednisone, leading to improvement in skin lesions. This case highlights the importance of recognizing cutaneous manifestations as potential indicators of systemic sarcoidosis, and the need for a comprehensive, multidisciplinary approach to diagnosis and management.

## Introduction

Sarcoidosis is a multisystem inflammatory disease characterized by the infiltration of non-caseating granulomas into areas such as the skin, kidneys, lungs, and lymph nodes. Patients commonly exhibit a flu-like prodrome as a result of an increased inflammatory state. Cutaneous lesions from sarcoidosis occur in about 25% of patients and commonly include papules, nodules, plaques, and infiltrated scars [1]. The diagnosis of sarcoidosis is challenging, necessitating histological evidence of non-caseating granulomas, and an extensive work-up of sarcoidosis is integral due to its systemic nature. Non-caseating granulomas form as a result of ongoing inflammation that causes an accumulation of T-cells and macrophages, which then secrete cytokines and tumor necrosis factor-α [2]. While a precise causative agent of sarcoidosis has yet to be identified, studies suggest that it may be the result of an exaggerated immune response in a genetically susceptible individual to an undefined antigen [3]. The antigen could possibly be from environmental factors, microbes, or other sources. Regardless, there is strong evidence of a genetic predisposition to developing sarcoidosis, as heritability was shown to be approximately 66% in a twin study [4,5].

Over 60% of deaths in sarcoidosis patients worldwide are due to pulmonary involvement [6]. Imaging studies, such as chest X-rays and CT scans, aid in evaluating lung complications. Additionally, laboratory tests, such as angiotensin-converting enzyme (ACE) levels and inflammatory markers, help monitor disease activity [7]. Up to 40% of pulmonary sarcoidosis patients progress to Stage IV disease, at which point there is minimal chance for spontaneous resolution [8].

Identifying sarcoidosis is challenging due to the nature of its nonspecific symptoms and diverse clinical manifestations. Many other diseases present with similar clinical, radiologic, and histologic findings such as tuberculosis, histoplasmosis, malignancy, and autoimmune conditions [9]. This case highlights an atypical presentation of sarcoidosis with cutaneous, pulmonary, and renal involvement, emphasizing the need for a thorough evaluation. While sarcoidosis itself is not uncommon, it brings forth the importance of diagnostic workup, given that this patient with a nonspecific, asymptomatic rash was found to have diffuse multi-organ involvement of a major disease.

## Case presentation

**Figure 1:**
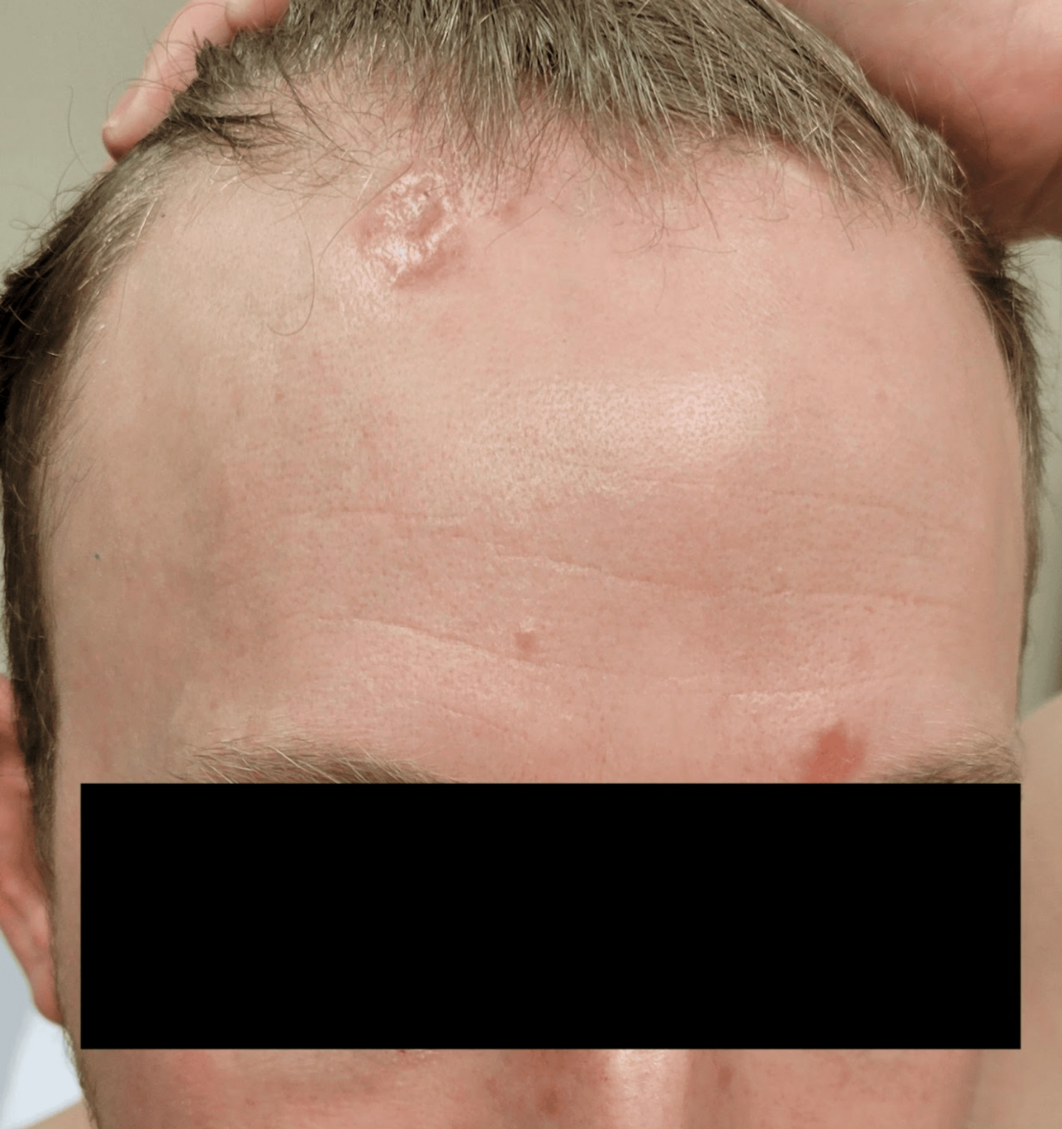
Plaques on the forehead of the patient with sarcoidosis

**Figure 2:**
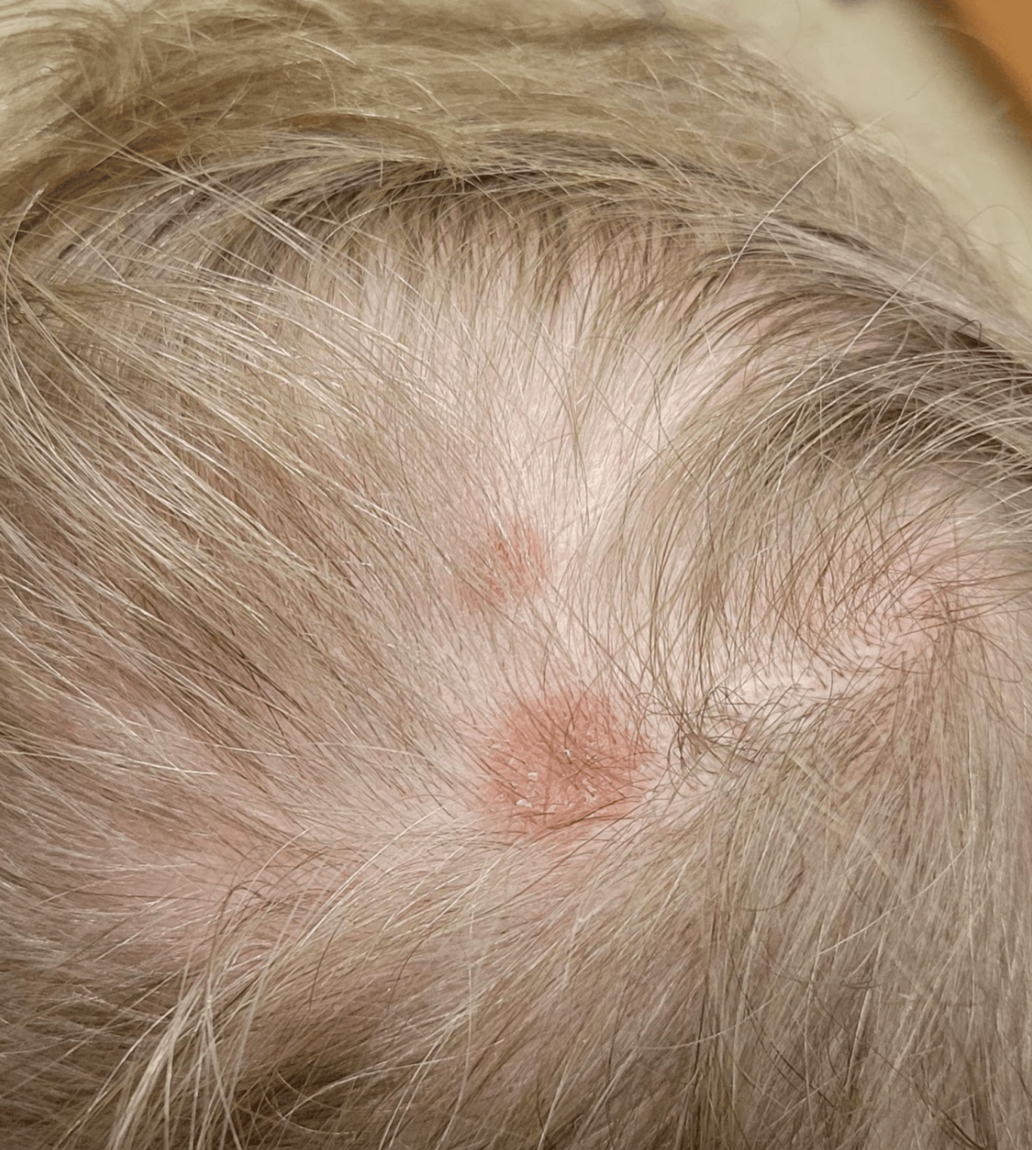
Plaques on the scalp of the patient with sarcoidosis

**Figure 3:**
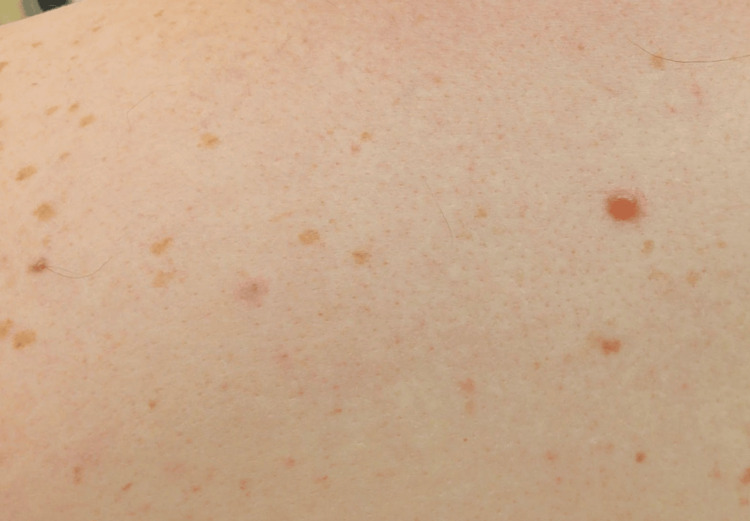
Reddish-brown papules on the back of the patient with sarcoidosis

A 29-year-old male patient with a history of recurrent kidney stones presented to dermatology with a one-year history of asymptomatic reddish-brown papules and plaques on his forehead, scalp, back, left arm, left thigh, and right foot. He also reported progressive dyspnea on exertion and a history of hematuria and hypercalciuria. Physical examination was notable for the aforementioned plaques (Figures 1, 2) and reddish-brown dermal papules (Figure 3), as well as bilateral cervical lymphadenopathy. Upon presentation to Dermatology, the skin lesions were biopsied and sent to Surgical Pathology for further inquiry.

Laboratory workup revealed an elevated ACE level, while complete blood count (CBC) and comprehensive metabolic panel (CMP) were unremarkable (Table 1).

**Table 1:** Laboratory findings of the patient with sarcoidosis BUN: blood urea nitrogen, ALT: alanine transaminase, AST: aspartate transaminase

Test	Results	References
White blood cells (k/uL)	6.20	4.00 – 11.00
Hematocrit (%)	43.1	40.0 – 52.0
Platelet count (k/uL)	260	150 – 450
Glucose (mg/dL)	75	74 – 99
Sodium (mg/dL)	136	136 – 145
Potassium (mg/dL)	4.2	3.4 – 4.8
Chloride (mg/dL)	98	98 – 107
BUN (mg/dL)	18	9 – 21
Creatinine (mg/dL)	1.1	0.7 – 1.3
Calcium (mg/dL)	10.0	8.5 – 10.5
Albumin (g/dL)	4.0	3.2 – 5.2
Total bilirubin	0.7	0.3 – 1.2
ALT (U/L)	25	< 55
AST (U/L)	28	< 35
Angiotensin-converting enzyme (U/L)	211	16 – 85

**Figure 4:**
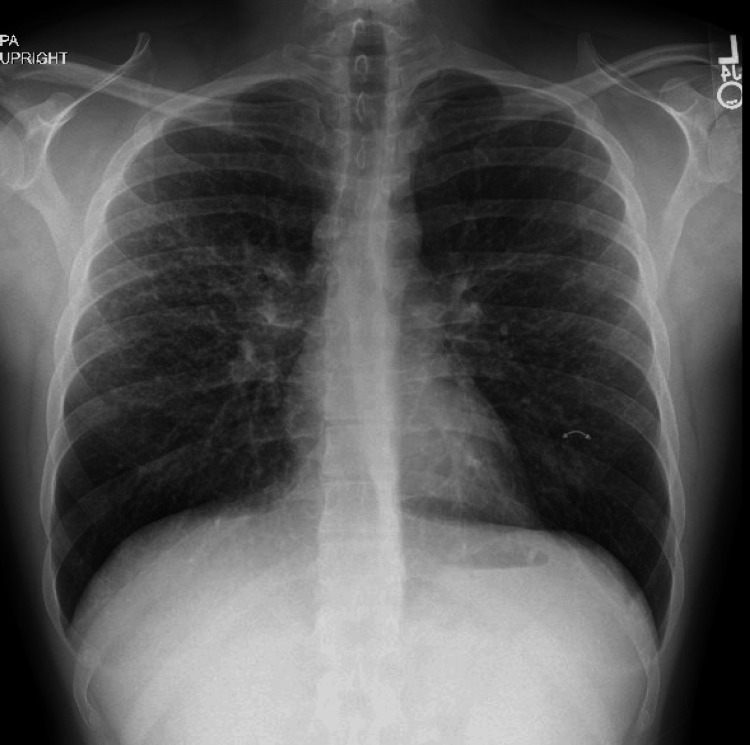
Chest X-ray of the patient suggesting diffuse pulmonary sarcoidosis

Chest X-ray findings were suggestive of diffuse pulmonary sarcoidosis (Figure 4). Classically, these findings include bilateral hilar lymphadenopathy and pulmonary fibrosis. Given his history of recurrent nephrolithiasis, sarcoid-related hypercalciuria was suspected. He was referred to Pulmonology and Urology for further management and initiated on hydroxychloroquine and prednisone, leading to improvement in his skin lesions at follow-up.

## Discussion

This case illustrates the diagnostic challenges of sarcoidosis and highlights the importance of cutaneous findings in recognizing systemic disease. The presence of cutaneous sarcoidosis in conjunction with pulmonary symptoms and recurrent kidney stones suggested a more extensive disease process, necessitating a comprehensive workup. The biopsy findings, along with elevated ACE levels and suggestive imaging, supported the diagnosis. ACE levels may be elevated in up to 75% of untreated patients; however, they lack specificity and have not been shown to correlate with disease severity [10,11]. While limited in clinical utility, it remains useful in possibly identifying new sarcoidosis.

Cutaneous sarcoidosis often presents in varied forms, including papules, plaques, and nodules, which can mimic other dermatologic conditions such as lupus, granuloma annulare, or infectious diseases. Histologic confirmation of non-caseating granulomas is essential to establish the diagnosis, and special stains must be performed to exclude infectious etiologies, particularly mycobacterial and fungal infections. In this patient, the periodic acid-Schiff-diastase (PASd), acid-fast bacillus (AFB), and Fite stains were negative for microorganisms such as *Nocardia sp.*,* Mycobacterium lepra*,and* Mycobacterium tuberculosis *[12].

Pulmonary involvement is the most common manifestation of sarcoidosis and the leading cause of sarcoidosis-related mortality [6]. While no treatment is indicated for asymptomatic stage I or II sarcoidosis due to high rates of spontaneous resolution, those with symptomatic or progressive stage II or III pulmonary disease require systemic corticosteroid treatment [9]. Limited data reveals improvement in symptoms and lung function in these patients; however, no effect has been noted on mortality or long-term outcomes. Severe pulmonary sarcoidosis warrants referral for possible lung transplantation.

The presence of hypercalciuria and recurrent kidney stones in this patient suggested extra-pulmonary involvement, as sarcoidosis is known to cause dysregulated vitamin D metabolism, leading to increased calcium absorption and nephrolithiasis [7].

The patient was treated with a combination of hydroxychloroquine and prednisone, a regimen often used for cutaneous and systemic sarcoidosis. Corticosteroids remain the mainstay of therapy, particularly in cases with significant organ involvement [8]. Hydroxychloroquine, an antimalarial agent, is sometimes added to cutaneous sarcoidosis due to its anti-inflammatory properties and steroid-sparing potential [13]. This patient was able to have symptomatic improvement on treatment after a comprehensive work-up that was initiated from the identification of his cutaneous lesions. Given the risk of disease progression and the need for long-term monitoring, continued multidisciplinary follow-up is essential in this case.

## Conclusions

This case is noteworthy in that it highlights the importance of recognizing cutaneous sarcoidosis as a potential early marker of systemic disease. The extent of involvement (hyperuricemia, nephrolithiasis, and diffuse pulmonary fibrosis) is vast, particularly for someone presenting with merely a nonspecific rash, and it is crucial to make the diagnosis as early as possible to initiate management. Dermatologic findings may be the first clue to an underlying multi-system disorder, emphasizing the need for more thorough evaluation and early intervention. Physicians should maintain a high degree of suspicion when encountering unexplained skin lesions, particularly in patients with additional systemic symptoms, such as the patient in this case.

The management of sarcoidosis requires an individualized approach, considering disease severity, organ involvement, and response to therapy. A multidisciplinary approach, involving dermatologists, pulmonologists, nephrologists, and other specialists, is critical to ensuring a timely diagnosis and treatment. By recognizing and addressing cutaneous sarcoidosis in the broader context of systemic disease, physicians can facilitate early intervention, improve patient outcomes, and potentially reduce long-term morbidity and mortality associated with sarcoidosis.
